# Extracellular Vesicles Mediate Anti-Oxidative Response—In Vitro Study in the Ocular Drainage System

**DOI:** 10.3390/ijms21176105

**Published:** 2020-08-25

**Authors:** Natalie Lerner, Itay Chen, Sofia Schreiber-Avissar, Elie Beit-Yannai

**Affiliations:** Clinical Biochemistry and Pharmacology Department, Ben-Gurion University of the Negev, Beer-Sheva 84105, Israel; karpnko@post.bgu.ac.il (N.L.); itay.timrat@gmail.com (I.C.); sofia@bgu.ac.il (S.S.-A.)

**Keywords:** extracellular vesicles, exosomes, primary open-angle glaucoma, trabecular meshwork, non-pigmented ciliary epithelium, OS, Nrf2-Keap1

## Abstract

The importance of extracellular vesicles (EVs) as signaling mediators has been emphasized for several pathways with only limited data regarding their role as protective messages during oxidative stress (OS). The ocular drainage system is unique by being continuously exposed to OS and having a one-way flow of the aqueous humor carrying EVs taking role in glaucoma disease. Here, we aimed to examine the ability of EVs derived from the non-pigmented ciliary epithelium (NPCE)—the aqueous humor producing cells exposed to OS—to deliver protecting messages to the trabecular meshwork (TM)—the aqueous humor draining cells—a process with significance to the pathophysiology of glaucoma disease. EVs extracted from media of NPCE cells exposed to non-lethal OS and their unstressed control were incubated with TM cells. The effects of EVs derived from oxidative stressed cells on the activation of the nuclear factor erythroid 2-related factor 2-Kelch-like ECH-associated protein 1 (Nrf2-Keap1), a major OS pathway, and of the Wnt pathway, known for its role in primary open-angle glaucoma, were evaluated. EVs derived from oxidized NPCE cells significantly protected TM cells from direct OS. The TM cells uptake of EVs from oxidized NPCE and their cytosolic Nrf2 levels were significantly higher at 8 h post-exposure. EVs derived from oxidized NPCE cells significantly attenuated Wnt protein expression in TM cells and activated major antioxidant genes as measured by qRT-PCR. TM cells exposed to EVs derived from oxidized NPCE cells exhibited significantly lower OS and higher super oxide dismutase and catalase activity. Finally, we were able to show that carbonylated proteins and products of oxidized protein are presented in significantly higher levels in EVs derived from oxidized NPCE cells, supporting their suggested role in the signaling process. We hypothesize that these findings may have implications beyond understanding the pathophysiology of glaucoma disease and that transmitting signals that activate the antioxidant system in target cells represent a broad response common to many tissues communication.

## 1. Introduction

Communication mediated by extracellular vesicles (EVs) has been shown to be part of tissue and organ general homeostasis and plays a significant role during pathologies. Oxidative stress (OS) due to imbalance between cell oxidant exposure and cell antioxidant capacity plays a pivotal role in determining cell fate. It is accepted among the scientific community that redox balance is one of the tightly controlled homeostasis parameters resembling physiological pH levels control. Local changes in OS are responded by corresponding changes in antioxidant capacity of the cells and tissues. The cellular defense mechanisms against OS include low molecular weight antioxidants and antioxidant enzymes. Cells are continuously exposed to OS by reactive oxygen species (ROS) leakage from the mitochondrial cellular respiration. Some degree of pro-OS is required to maintain physiological homeostasis, but uncontrolled elevated oxidative conditions might be cell harmful. One of the key redox regulating systems allowing cells to respond to OS is the Nrf2-Keap1 pathway. Nrf2 is an intracellular transcription factor that regulates the expression of genes encoding antioxidant enzymes, low molecular weight antioxidants, anti-apoptotic proteins, and drug transporters. Under normal condition, Nrf2 is usually degraded in the cytoplasm by interaction with Keap1 inhibitor as an adaptor for ubiquitination factors. However, high amount of ROS activates tyrosine kinases to dissociate Nrf2-Keap1 complex, Nrf2 translocate to the nucleus where a coordinated activation of cytoprotective gene expression takes place [1].

OS has been implicated in the major sight deteriorating diseases namely: aged macular degeneration, glaucoma, and cataracts. Glaucoma and cataracts are directly linked to OS morphologic and to physiologic alterations in the Aqueous humor (AqH) pathway in aging and glaucoma [2]. Continuous exposure to UV light leads to excessive production of ROS that was attributed to cataracts [3] and primary open-angle glaucoma (POAG) [4]. The outer segment is partially protected by antioxidant enzymes and low molecular weight antioxidant presented in the AqH and in the ocular drainage tissues [5,6,7]. Changes in the AqH reducing capacity were reported in a rabbit model of POAG [8,9].

Glaucoma is a heterogeneous group of diseases, characterized by retinal and optic nerve degeneration, resulting in progressive loss of visual field and irreversible blindness if left untreated [10]. POAG is a leading cause of blindness with no known cure [11]. AqH is the optically clear fluid supplying the avascular tissues with oxygen, nutrients, antioxidants as well as carrying out waist products. AqH was reported to mediate mitogen-activated protein kinase signals, phosphatase and matrix metalloproteinases activation in TM cells [12,13]. AqH is produced by the non-pigmented ciliary epithelium (NPCE) of the ciliary processes and it has been hypothesized to convey signaling message between the ciliary epithelium tissue and the TM [14]. The cellular mechanism responsible for cell-cell communication has remained an enigma.

Recently, we were able to show that EVs released by the NPCE can modulate the Wnt/β-catenin signaling pathway in the TM cells in vitro [15]. EVs known as exosomes are nano-sized lipid bilayer vesicles that are released from cells upon fusion of an intermediate endocytic compartment, the multivesicular body (MVB), with the plasma membrane. This liberates intraluminal vesicles into the extracellular milieu. EV cargo includes nucleic acid content such as small RNAs (mRNA, miRNA, tRNA, rRNA and other) and ssDNA [16]. In addition, many proteins and lipids [17] were found in EVs, some of them common to many EVs and other unique or related to physiological conditions. Extensive research during recent years revealed that EVs participate in many biological processes, for example: tissue signaling [18], metastasis spreading [19], immune response [20] and wound healing [21]. Recently, Klingeborn et al. published a comprehensive review of EV roles in normal and diseased eye [22].

The ability of EVs to carry protective signals following OS has been described [23,24,25]. Recently, R.M. Ramirez et al. summarized the role of microvesicles in ROS scavengers and producers [24]. In the ocular system, a couple of papers described the consequence of retinal pigment epithelium cells exposure to OS by EV-mediated signals [26,27]. Combining the present knowledge about the ocular outer segment exposure to OS in POAG and the role of EVs in signaling communication, we hypothesized that EVs derived from oxidative stressed ocular tissues might have a role in the ocular drainage system by delivering stress signals. Therefore, we investigated whether exposure of TM cells to EVs produced by NPCE cells under OS conditions can result in TM biology changes inducing cell-protective mechanisms.

## 2. Results

### 2.1. Cell Viability of AAPH-Treated NPCE Cells

**Figure 1:**
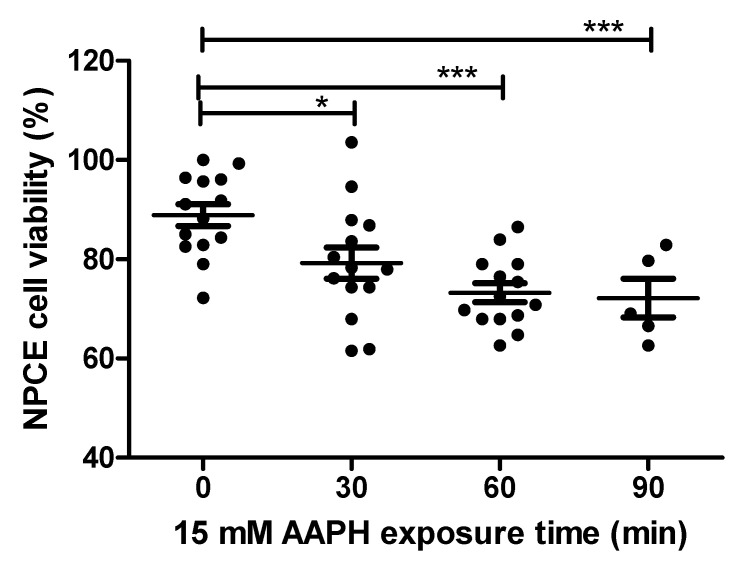
Cell viability of AAPH-treated NPCE cells. Cell viability was measured by MTT assay in NPCE cells at 30, 60, 90 min after 15 mM AAPH treatment. Data are expressed as percent viability and represented by mean ± SEM. Multiple group comparisons were performed using one-way analysis of variance (ANOVA) followed by the post hoc Tukey’s test, and any difference in comparison with untreated NPCE cells was considered significant when * *p* < 0.05 or *** *p* < 0.001.

NPCE cells were exposed to OS in order to find the conditions for significantly reduced NPCE cells viability to such a degree that still enables processes related to the production and release of EVs to remain significantly unaffected. AAPH (2,2′-Azobis(2-methylpropionamidine) dihydrochloride) a super oxide generator was used to induce OS in NPCE cells. These cells were treated with two concentrations (10 mM and 15 mM) of AAPH at different incubation times and the medium was replaced 24 h thereafter. Cytotoxicity was measured by MTT assay. The results depicted in Figure 1 demonstrate a time-dependent reduction in cell survival. 15 mM AAPH for 90 min, which resulted in a significant 20% viability reduction (*p* < 0.001), was chosen for the next experiments (Figure 1).

### 2.2. EV Size from OS Exposed NPCE Cell 

Literature data suggested that oxidative stressed cell responded by changes in the amount of secreted EVs without changes in EV sizes [27]. In our research we found that the EVs from AAPH exposed NPCE cells have the same size as naïve NPCE cells derived EVs [105 nm and 104 nm respectively]. The in vitro conditions in which the cells were grown and the EVs extracted do not allow accurate determination of the secreted EVs concentrations and whether there is a change in their secretion rate following exposure to AAPH.

### 2.3. Carbonylated Protein Presence in Oxidized NPCE-Derived EVs

**Figure 2:**
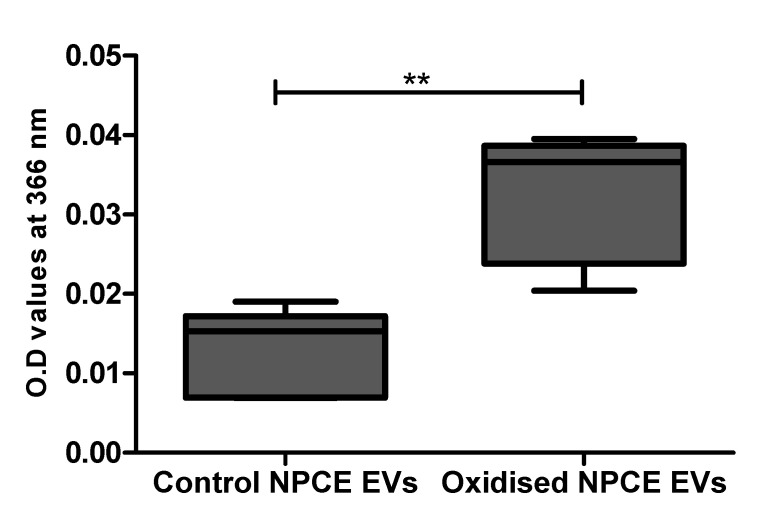
Carbonylated protein presence in oxidized NPCE-derived EVs. Carbonylated proteins in control and oxidized NPCE cells (15 mM AAPH, 90 min) derived EVs were measured at 366 nm using the DNPH method. 1.28 × 10^11^ particle/mL equal to 0.30 mg proteins/mL were used. Data are represented by mean ± SD. Comparisons were performed by two tails *t*-test, and any difference in comparison were significant with ** *p* < 0.01.

EVs extracted from the condition media of oxidative exposed AAPH as described in the method paragraph, were analyzed by spectrophotometer at 366 nm using the DNPHDNPH (2,4-dinitrophenylhydrazine) method for carbonylated protein detection. A preliminary study in our lab on AAPH oxidized TM cells suggested the minimal exosome protein concentration needed for reliable results by this method are 0.1–0.5 mg protein (Appendix A). NPCE EVs 1.28 × 10^11^ particle/mL = 0.30 mg proteins/mL were compared to control NPCE EVs for carbonylated protein content. A significant increase in carbonyl content was found in oxidized NPCE EVs (Figure 2).

### 2.4. TM Cells Viability After Co-Culture with NPCE EVs

**Figure 3:**
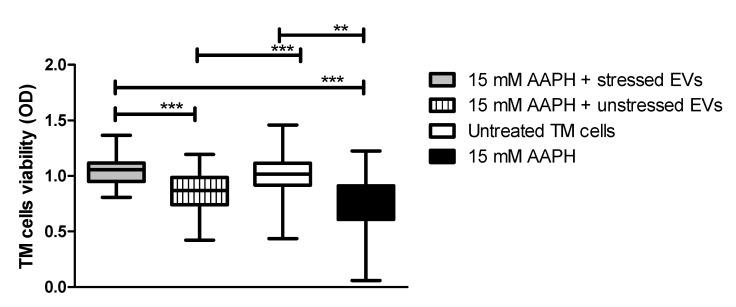
AAPH-treated TM cell viability after co-culture with NPCE EVs. TM cells were pretreated with AAPH (15 mM) for 1.5 h at 37 °C. Afterwards, the medium was removed and either unstressed NPCE EVs or stressed NPCE EVs were added to the TM culture, and the cells were cultured for another 24 h. Effect of NPCE EVs on the TM cell damage induced by AAPH was determined by MTT assay and compared to the TM cells under normal or OS conditions. Data from three independent experiments are represented by means ± SEM. Multiple group comparisons were performed using one-way ANOVA followed by the post hoc Tukey’s test, where ** *p* < 0.01, *** *p* < 0.001.

It was previously demonstrated that human cells exposed to OS conditions react to stress by activating antioxidant molecules, which can be released through EVs that provide recipient cells with a resistance against OS [28]. Based on these findings, we determined whether treatment of TM cells with NPCE EVs provide protection from apoptosis induced by OS. TM cells were directly exposed to oxidative stress by 15 mM AAPH for 90 min and afterwards TM cells were co-cultured with EVs derived from oxidative stressed NPCE cells (EVs-OS) or from NPCE cells without stress (EVs-N). Untreated TM cells or TM cells treated with 15 mM AAPH only without EV exposure (NT TM) served as controls. TM cell viability following EVs treatment was determined by MTT assay. As can be seen in Figure 3 in TM challenged by oxidative stress (OS TM) a significant reduction in the cell viability was observed (*p* < 0.001) relative to untreated control cells. EVs-N could not avoid TM cell viability reduction induced by AAPH treatment. When EVs-OS were added to OS TM, the TM cell viability remained similar to that of untreated TM cells. These results indicate that EVs-OS protect TM cells from OS-induced death.

### 2.5. The Effect of NPCE-Derived EVs on Nrf2 Levels in TM Cells

**Figure 4:**
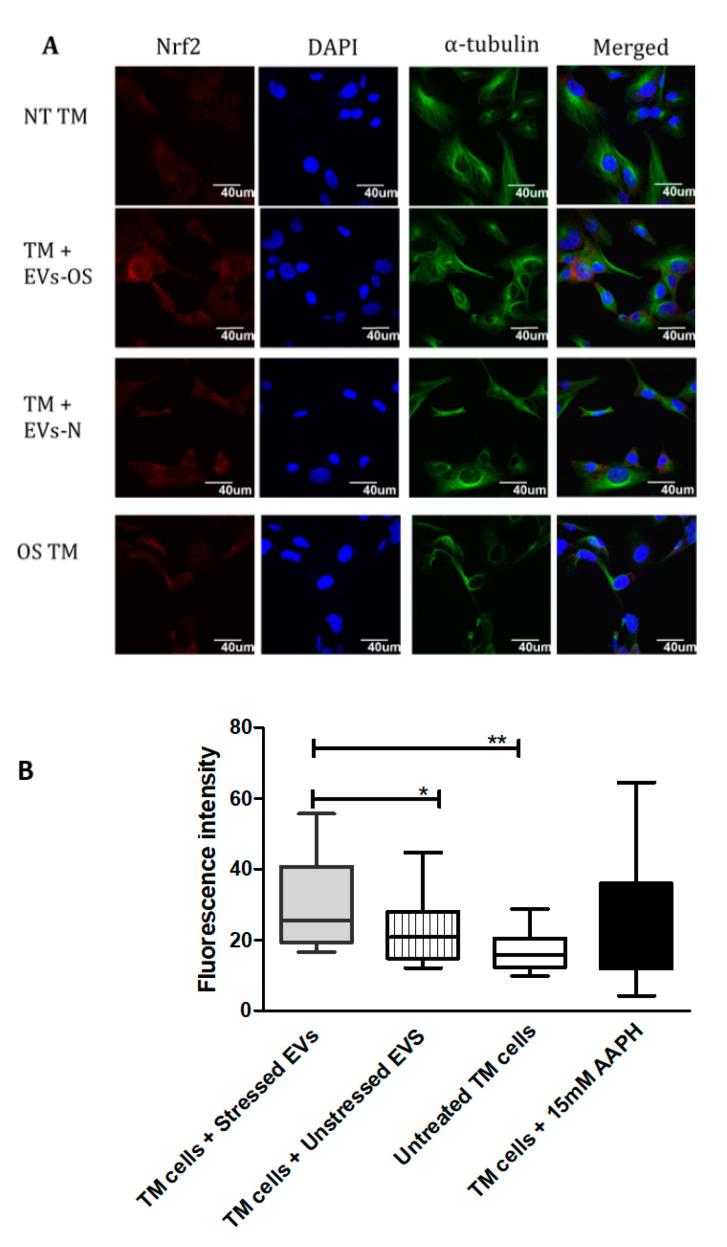
Immunocytochemical detection of Nrf2 in TM cells under normal or stress conditions following exposure to NPCE-derived EVs. (**A**) Representative confocal images of Nrf2 staining in untreated TM cells or TM cells co-incubated with NPCE EVs released under normal or OS conditions, or TM subjected to OS with 15 mM AAPH for 1.5 h. TM cells were co-stained with antibody against the cytoskeleton marker α-tubulin (green) and nuclei were stained with DAPI (blue) (**B**). Quantification of Nrf2 expression level. Results are displayed as mean ± SEM of fluorescent intensity/number of cells, where * *p* < 0.05, ** *p* < 0.01 in one-way ANOVA with post hoc Tukey’s test.

**Figure 5:**
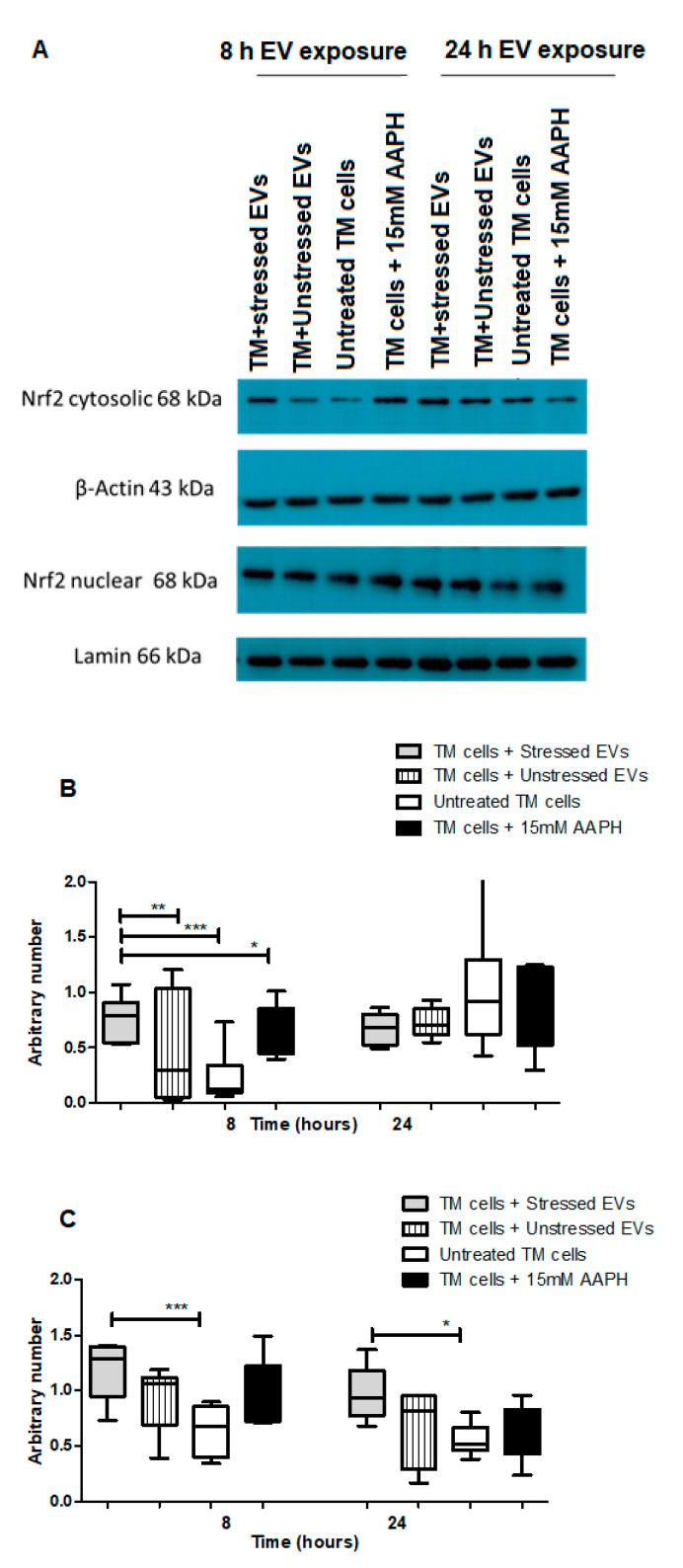
Western blot analysis of Nrf2 expression in TM cells in response to NPCE EVs under basal or OS conditions. (**A**) Representative Immunoblots of Nrf2 in nucleus and cytosol of either untreated TM cells or TM cells treated with 15 mM AAPH for 1.5 h or TM cells co-incubated for 8 or 24 h with NPCE EVs released under normal or stressed conditions. Cytosolic and nuclear proteins were separated by centrifugation procedure, resolved by SDS-PAGE and blotted onto PVDF membrane. Nrf2 was visualized with polyclonal antibody. Lamin and β-actin were used as loading controls (**B**) Densitometry analyses of cytosolic Nrf2 and (**C**) Nuclear Nrf2. Data are presented as the mean ± SEM, where * *p* < 0.05, ** *p* < 0.01, *** *p* < 0.001 in two-way ANOVA with post hoc Tukey’s test.

Nrf2-Keap1-ARM signaling plays a significant role in cell protection from exogenous and endogenous stresses. In general, the transcription factor Nrf2 is bound to Keap1 in the cytoplasm allowing basal expression of Nrf2 regulated gene. Upon cell exposure to different stressors producing OS, Nrf2 Keap1 bond is broken allowing Nrf2 to translocate to the nucleus and activate the expression of cyto-protective genes [29]. We tested the ability of NPCE EVs to modulate Nrf2 expression in TM cells using immunohistochemistry and western blot analysis. Results showed that treatment with EVs-OS clearly increased the Nrf2 staining in the TM cytoplasm and nucleus. A trend of increase in Nrf2 expression was detected when TM cells were directly exposed to AAPH and when control NPCE EVs were added (Figure 4A,B). Western blot results showed significant increase in the levels of cytoplasmic Nrf2 for TM cells co-cultured with EVs-OS and cells treated with 15 mM AAPH, compared to untreated control cells or OS TM cells exposed to EVs-N (Figure 5B,C). Although in nuclear fraction we observed higher expression of Nrf2 protein, in TM cells co-culture with either EVs-OS or EVs-N under oxidative stress conditions (Figure 5B,C).

### 2.6. The Effect of NPCE-Derived EVs on Wnt Proteins Levels Under Stress Conditions

**Figure 6:**
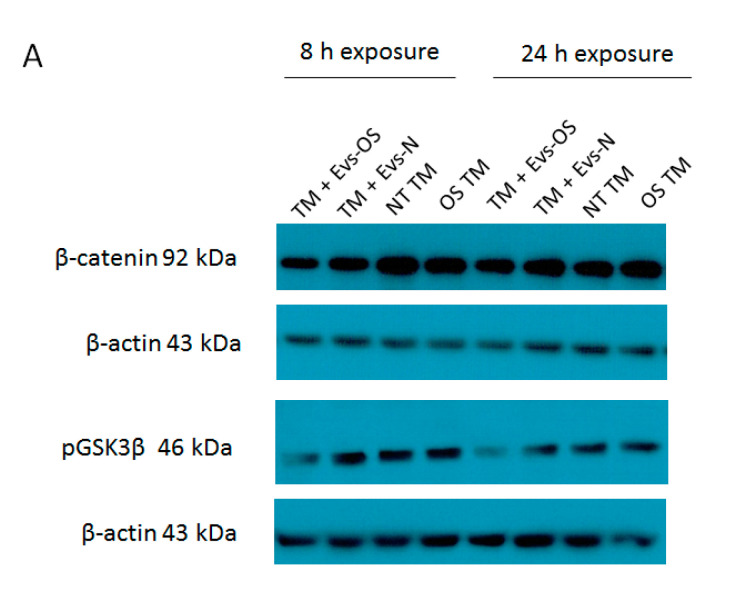

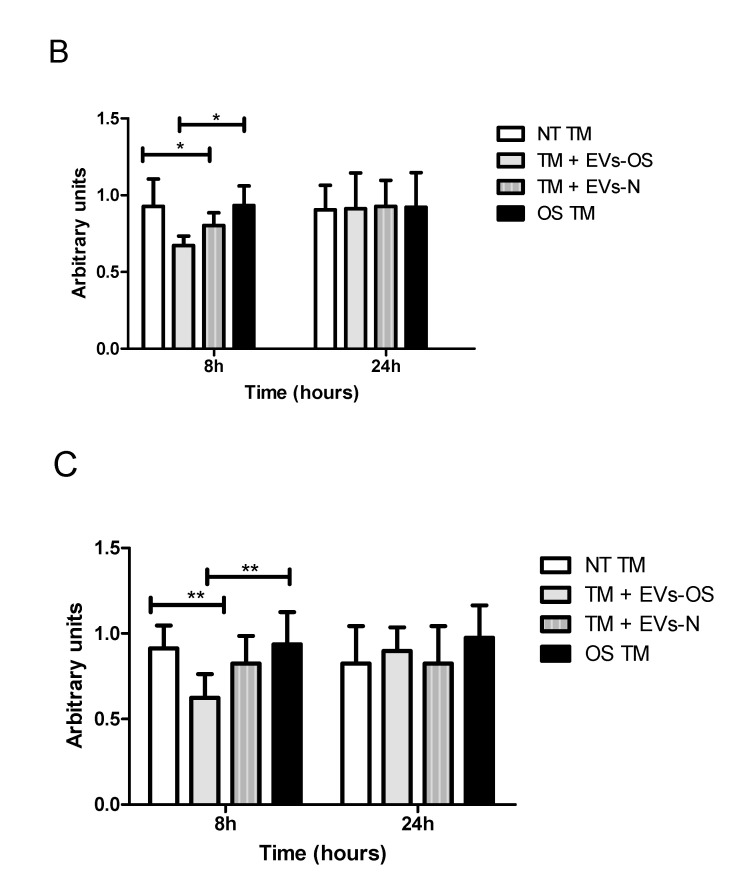
Wnt protein expression under normal or stress conditions following exposure to NPCE-derived EVs. Protein expression levels of β-catenin and p-GSK3β in TM cells under the treatment of NPCE exosomes. (**A**) Representative Western blots showing protein expression of β-catenin and p-GSK3β in cell lysates from TM cells treated with NPCE stressed or normal exosomes for 8, 24 h, or 15 mM AAPH for 1.5 h. Quantification of (**B**) β-catenin and (**C**) p-GSK3β protein levels from three independent experiments (*n* = 3). β-actin was used as internal loading control. Data are presented as the mean ± SEM, where * *p* < 0.05, ** *p* < 0.01 in two-way ANOVA with post hoc Bonferroni test.

Oxidative stress was shown to antagonize Wnt signaling [30] and a previous study in our lab demonstrated the ability of NPCE derived EVs to attenuate Wnt signaling pathway in TM cells [31]. Two key proteins affected were p-GSK3β and β-catenin. Here we examined these protein expressions in TM cell under direct OS and following exposure to EVs derived from NPCE cells exposed to OS. The results show that expression levels of p-GSK3β and β-catenin were reduced in the TM cells co-cultured either with EVs-OS or EVs-N than untreated TM and OS TM at 8 h. However, pretreatment with the EVs-OS more effectively attenuated the expression of Wnt proteins compared to untreated NPCE derived EVs (Figure 6B,C).

### 2.7. Effect of the NPCE EVs Released Under Normal or Oxidative Stress Conditions on the Expression of Anti-Oxidative Genes in TM Cells

**Figure 7:**
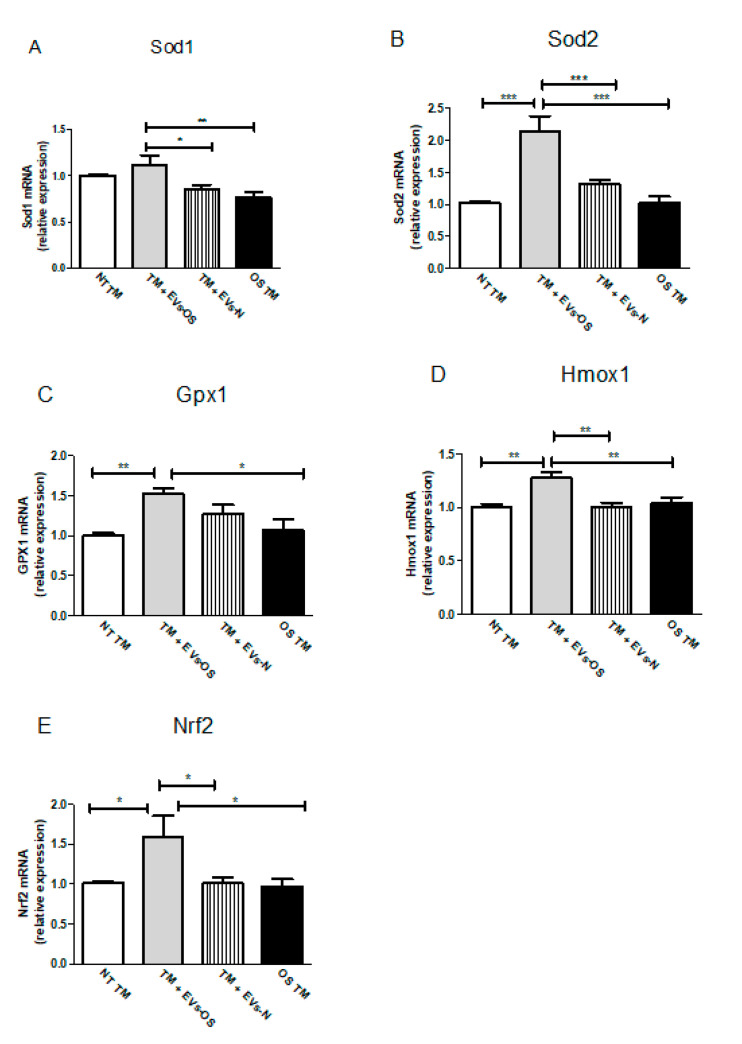
Assessment of oxidative stress-related genes in TM cells. Total RNA was isolated from untreated TM cells or TM cells treated for 1.5 h with 15 mM AAPH, or TM co-incubated with either stressed or normal NPCE EVs, and quantitative real-time PCR was performed to measure the level of Sod1, Sod2, Gpx1, Hmox1, and Nrf2. The results were normalized to 18sRNA. Bars represent mean ± SEM of three independent experiments, where * *p* < 0.05, ** *p* < 0.01, *** *p* < 0.001 in one-way ANOVA with post hoc Tukey’s test.

Changes in Nrf2 expression and increased Nrf2 levels are expected to increase cell survival response partially through anti-oxidative genes expression. Following TM OS and TM incubation with NPCE EVs and oxidative stressed NPCE derived EVs major TM Nrf2 and its downstream antioxidant genes expressions were analyzed by qRT-PCR. As shown in Figure 7, the expression levels of all five examined anti-oxidative genes (Sod1, Sod2, Gpx1, Hmox1, and Nrf2) were significantly up-regulated in the TM cells co-cultured with EVs-OS compared to untreated TM or TM exposed either to EVs-N or to direct oxidative stress with 15 mM AAPH.

### 2.8. NPCE Exosomal Protective Effect from the AAPH-Induced OS

**Figure 8:**
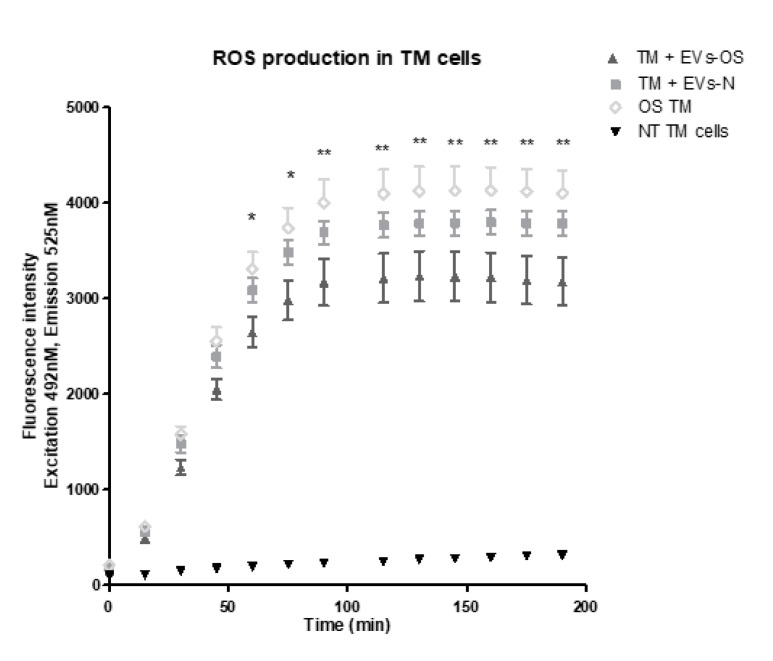
Kinetic estimation of AAPH-induced ROS generation in TM cells following incubation with normal or stressed NPCE EVs. TM cells were pretreated with normal or stressed NPCE exosomes for 24 h, and then rinsed with Phosphate buffer saline. Subsequently, the pretreated cells were incubated with DCFDA (2′,7′-dichlorofluorescin diacetate) for another 1 h at 37 °C. This was followed by the washing step and addition of either PBS or AAPH (150 µM). ROS amounts in TM cells were quantified with or without NPCE exosomes pretreatments and results are presented as mean fluorescence intensity ± SEM from three independent experiments. Fluorescence was determined at 15 min intervals over a period of 3 h. The asterisks indicate significant differences between the untreated TM cells and TM treated with NPCE stressed EVs. * *p* < 0.05, ** *p* < 0.01, in one-way ANOVA with post hoc Tukey’s test.

We next examined the effects of NPCE EVs on ROS formation using DCF-DA reagent. Our findings revealed that both EVs-N and EVs-OS reduced the accumulation of 15 mM AAPH-triggered ROS in TM cells. However, TM incubated with EVs-OS showed a more marked effect on ROS reduction compared to EVs-N treatment (Figure 8). These results suggest that EVs-OS have protective effect against AAPH-induced oxidative damage.

### 2.9. NPCE Effect on the Activity of Catalase (CAT) and Super Oxide Dismutase (SOD) Under Normal or OS Conditions

**Figure 9:**
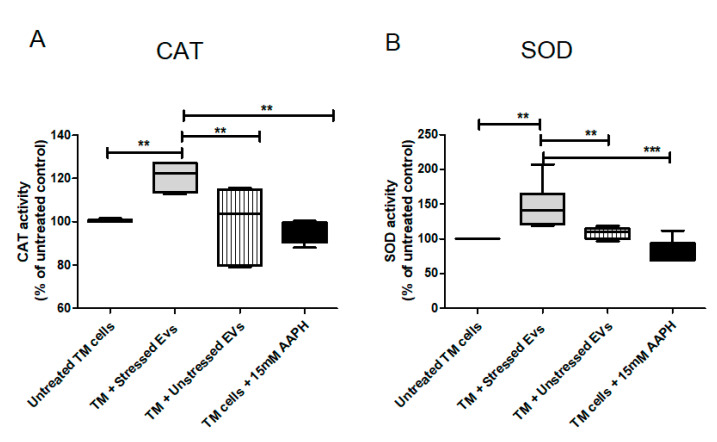
Effects of pretreatments with NPCE EVs on SOD and CAT enzyme activities in TM cells. Proteins from TM cells were extracted 24 h post-NPCE exosomes treatment and used as source of SOD or CAT. Quantification of the antioxidant activity of (**A**) CAT and (**B**) SOD in TM cells. The results are mean±SEM of 4 different experiments. Significance tested by one-way ANOVA and Tukey’s post hoc analysis. Levels of significance are marked with symbols ** *p* < 0.01, *** *p* < 0.001.

CAT is one of the most important antioxidant enzymes, present in almost all aerobically respiring organisms. The main function of CAT is decomposition of hydrogen peroxide into water and molecular oxygen. The TM is a metabolically active tissue that has been found to contain key enzymes involved in protecting against OS [32]. Increased CAT activity following Nrf2-Keap1 induction was reported in many papers/reports. In our model, NPCE cell exposed to OS released EVs with the ability to significantly induce CAT activity in TM cells. From the results shown in Figure 9A it was clear that CAT activity was higher by 50% in TM cells co-cultured with EVs-OS relative to CAT activity measured following exposure of TM cells to direct oxidative stress (*p* < 0.001) or exposure of TM cells to EVs-N (*p* < 0.01) or untreated TM cells (*p* < 0.01). SOD is one of the most important defense enzymes, present in the TM and has been shown to decline in an age-dependent way in normal human TM [33]. As clearly shown in Figure 9B, treatment of TM cells with NPCE EVs produced a significant increase of 20% in SOD activity compared to untreated TM (*p* < 0.01), oxidative stressed TM (*p* < 0.01) and TM exposed to normal NPCE EVs (*p* < 0.01). These results demonstrate the ability of NPCE EVs-OS to regulate the activity of CAT and SOD in TM cells.

## 3. Discussion

The understanding of pathological processes in POAG has deepened in recent years so that the involvement of OS in these processes is now well known. The ocular drainage system is exposed to OS [34] and the AqH and TM were found to have an antioxidant defense system composed of low molecular weight antioxidants and enzymes [33,34].

Limited publications have addressed the role of EVs as OS signal mediators. Eldh, and colleagues showed that EVs released from mast cells exposed to OS have the capacity to communicate a protective signal to recipient cells exposed to OS, and observed an attenuated loss of cell viability and changes in RNA content of EVs derived from oxidative stressed cells [35]. EV cargo variation was reported under different stresses and included miRNA, mRNA and protein modification [36]. Recently, horizontal transfer of defense molecules from EVs to granulosa cells was demonstrated in vitro using bovine granulosa cells. The cells exposed to OS released EVs enriched with Nrf2 mRNA and candidate antioxidants. Subsequent co-incubation of these EVs with cultured cells could alter the cellular OS response [28]. In addition, damage to RNA from ultraviolet light, oxidation, can result in chemical modifications to nucleotide as well as RNA-RNA and RNA-protein crosslinking. In our hands, EVs as a communication mediator between cells in vitro suggest a protected way for either nucleotide-based message or proteins-based message to be transferred. The changes in EV cargo under changing physiological condition allow dynamic response upon the circumstances.

In the present study, oxidative stressed NPCE-derived EV incubation with their target, the TM cells, resulted in significant Nrf2 induction and downstream response, which is expressed by increased antioxidant genes and altered protein expression, increased CAT and SOD activity. When TM cells were treated with normal NPCE-derived EVs, none of these changes was found. Specifically, Nrf2 expression did not change, no nucleic translocation of Nrf2 was found. Oxidative gene expression was not affected as CAT and SOD activity did not change vs. control TM. All these together suggest that the exposure of the NPCE cells to OS turned on the machinery in the NPCE MVB that resulted in modified NPCE EVs carrying protective message to the TM cells.

Possible candidates for protective messages transferred by EVs might be any cargo component including specific miRNA and siRNA, proteins, and lipids. EV mRNA content differs between EVs derived from cells grown under different conditions: OS or UV light and normal conditions [34]. Eldh, et al. used a commercial kit to immunoblot-oxidized protein from their experiment and presented data that no change in carbonylated protein in EVs derived from MC/9 cells exposed to H_2_O_2_. In the present research, we employed the DNPH method using a commonly used spectral analysis of carbonylated protein. A preliminary experiment examining the lower threshold for the detection of oxidized proteins by this spectral method required relatively large amounts of vesicles. Nevertheless, it allowed us to state with certainty that these oxidized proteins are present on the membrane of the EVs derived from oxidative exposed cells and may participate in EV communicating protective signals. We hypothesized that oxidized lipid and nucleotide end products can also be found in these EVs.

Previous study in our lab shows some specificity of the NPCE-derived EVs toward the TM cells. The specificity was manifested by higher NPCE-derived EV uptake by TM cells [15]. It will be interesting to repeat this specificity assay using EVs derived from oxidative NPCE and look for OS signals effects have some specificity or can transfer these signals to other ocular tissues and cells, in a nonspecific manner.

The Nrf2-Keap1 pathway activation by oxidative stressed NPCE-derived EVs suggests a new way by which intra ocular pressure might be regulated. Previously, we reported that the canonical Wnt pathway in TM cells can be modulated by NPCE-derived EVs [15,31]. This was replicated in the present study as shown in Figure 5. When NPCE EVs were extracted from NPCE cells exposed to OS this Wnt signaling effect was partially diminished. We propose that under OS, similar to what happens in POAG patients, the general homeostasis regulation of the TM resistance to AqH drainage by NPCE EVs is altered. Changes occurring in the NPCE cells exposed to OS are translated to their EV cargo and even to surface protein expression. As a result, modified NPCE EVs carrying stress convey alert signals to TM cells. We can speculate that as happens in inflammation processes while acute inflammatory response has beneficial effect, the development of a chronic situation can eventually cause a physiological problem. Analogically, the modifications in EV cargo of oxidative exposed NPCE cells aim to deliver a protective message to TM cells. However, continuous exposure to OS might result in EV-mediated messages that are either not inducing enough protection or even turning to harmful messages. Volarevic et al. suggested that mesenchymal stem cells derived EVs have the therapeutic capacity for the treatment of eye diseases [37]. Other researchers presented different approaches, such as engineering EVs to deliver specific miRNAs to target cells [38] or modifying parent cell EV content by transfection [39]. The potential use of EVs or modified EVs for therapeutics and diagnosis is being uncovered during the last year. Understanding the role of EVs in OS mediated protection will allow interfering with the beneficial and harmful consequences of EVs transferred signals.

## 4. Materials and Methods

### 4.1. Cell Lines

TM cell line was generously donated by Alcon Research, Ltd. (Fort Worth, TX, USA) and cultured in Dulbecco’s modified Eagle’s medium (DMEM) containing 10% fetal calf serum (FBS), 2 mM l-glutamine, 100 IU/mL penicillin, and 100 μg/mL of streptomycin (all from Biological Industries, Kibbutz Beit Ha-Emek, Israel). A human NPCE cell line was kindly provided by Dr. M. Coca-Prados (Yale University, New Haven, CT, USA). NPCE cells were routinely maintained with the same supplements in DMEM medium supplemented with FBS pre-depleted of EVs by overnight ultracentrifugation at 100,000× *g*. All cell lines were grown in controlled environment of 5% CO_2_ and 95% relative humidity at 37 °C.

### 4.2. EV Extraction by Polyethylene Glycol (PEG) Precipitation

EV samples were purified from NPCE cells under both normal (EVs-N) and oxidative stress conditions (EVS-OS) according to a PEG (Cat# 89510, Sigma, St. Louis, MO, USA)-based isolation method [40,41] as previously described, with minor modifications. NPCE cells were plated at 5 million cells per 75 cm^2^ and after reaching 90% confluence, the cells were exposed for 1.5 h 2,2′-Azobis(2-methylpropionamidine) dihydrochloride (AAPH)compound (Cat# 440914, Sigma, St. Louis, MO, USA). NPCE cell-conditioned medium was aspirated and centrifuged at 1500× *g* for 10 min to remove cells, followed by filtration through PVDF filter (0.22 µm, Millipore, Billerica, MA, USA) to remove large cellular debris. Precipitation solution (50% PEG8000, 0.5 M NaCl in PBS) was added to the cleared conditioned medium (1:5 *v*/*v*, respectively), mixed by flicking the tube and incubated overnight at 4 °C. After incubation, the tubes were centrifuged at 1500× *g* for 30 min at 4 °C to acquire the pellet of EVs. The supernatant was discarded and pelleted EVs were dissolved in 500 µL PBS for further analysis. The EVs-N were isolated from non-treated (NT) NPCE cells following the same procedure as described above.

### 4.3. Tunable Resistive Pulse Sensing (TRPS)

The concentration of isolated EVs was determined by TRPS technique using qNano gold instrument (Izon Science, UK). EVs samples were diluted in PBS buffer containing 0.05% Tween-20 (Cat#P1379, Sigma, St. Louis, MO, USA) and passed through 0.22 µm filters to get rid of contaminating debris. 80 μL of electrolyte solution was added to bottom fluid cell and 40 µL of diluted EV suspension was dispensed into the top fluid cell. The voltage of 0.65 V was applied, and the pressure was set at 7 mbar. Measurements were performed with NP-150 nanopore membrane stretched to 47 mm. The system was calibrated by polystyrene beads at a concentration of 1 × 10^13^ beads/mL (114 nm; Izon Science) supplied by the qNano manufacture. A minimum of 500 translocation events for each sample were recorded and the data analysis was performed with qNano-IZON software. The characteristics of NPCE-derived EVs were found to be identical to those previously reported in detail [15].

### 4.4. Carbonyl Assay

Oxidative protein damage was quantified using the carbonyl. Briefly, 200 μL of extracted EVs at a concentration of 1.28 × 10^11^ particle/mL (=0.30 mg proteins/mL) was combined with 40 µL of 10 mM 2,4-dinitrophenylhydrazine (DNPH) and 2N HCl. Samples were incubated at room temperature under dark conditions and agitated every 15 min during a period of 60 min before being precipitated with 20% *v*/*v* trichloroacetic acid (TCA). Samples were then centrifuged for 5 min at 10,000× *g* to collect the precipitated protein. The pellet was washed with 200 L of 20% TCA. Subsequently, the precipitate was washed with 200 µL of a mixture of ethyl acetate and ethanol (1:1 *v*/*v*) to eliminate remaining DNPH. The sample was centrifuged, and the final precipitate was dissolved in 200 µL of 6 M guanidine hydrochloride and 50 mM phosphate buffer, and was then incubated for 25 minut at 37 °C. Lastly, the products were analyzed with a spectrophotometer at a wavelength of 366 nm [30].

### 4.5. MTT Assay

The MTT assay, using the 3-(4,5-dimethylthiazol-2-yl)-2,5-diphenyl tetrazolium bromide (MTT) reagent (Cat# M2128, Sigma, St. Louis, MO, USA) was chosen to determine the appropriate concentration of AAPH required to induce moderate OS in NPCE cells. The cells were seeded in 96-well plates at a density of 5 × 10^3^ cells/well at 37 °C in a humidified atmosphere with 5% CO_2_. Next day, the medium was removed and AAPH at different concentrations was added (10 and 15 mM) for various periods of time (30, 60, and 90 min). After incubation, cell survival was assayed by discarding the medium and adding 100 μL of medium containing 0.5 mg/mL MTT solution to each well to allow the MTT to be metabolized. After 4 h, 200 μL DMSO was added to dissolve formazan crystals. The absorbance of each sample was measured at 570 nm by a microplate reader (Model 680, Bio-Rad, Hercules, CA, USA). The effect of oxidative stressed EV released under normal conditions on TM cells viability following exposure to AAPH compound was further studied. TM cells (5 × 10^4^) were seeded in 96-well plates and grown for 24 h. The medium was removed, and the cells were incubated with DMEM medium in the presence or absence of 15 mM AAPH. One and half-hours later, the cells were washed with PBS and co-cultured with either EVS-N or EVS-OS for 24 h at a ratio of 1:100 (TM cells: EVs). Thereafter, MTT assay was performed to evaluate viability of TM cells.

### 4.6. Immunofluorescent Analysis of Nrf2 Expression

For visualization of Nrf2, TM cells were seeded at a density of 3 × 10^4^ cells/well in 24-well plates containing coverslips. Twenty-four hours later, cell medium was aspirated, and the cells were challenged for 1.5 h with 15 mM AAPH followed by incubation with EVS-N or EVS-OS for 24 h in DMEM medium supplemented with 10% EV-free fetal calf serum. Control cells were either treated with 15 mM AAPH (OS TM) or left untreated. The cells were then rinsed using 0.05% PBS/Tween 20, permeabilized using 0.5% PBS/Triton X-100 (Cat # X100, Sigma, St. Louis, MO, USA), fixed for 20 min with 4% paraformaldehyde at room temperature and blocked with 3% bovine serum albumin (BSA) for 30 min. Immunocytochemical staining was performed for Nrf2 and α-tubulin proteins by anti-human Nrf2 rabbit monoclonal antibody (1:200, ab62352, Abcam) and mouse anti-α-tubulin antibody (1:200, 625901, BioLegend). After overnight incubation at 4 °C with primary antibodies, the cells were washed and incubated for 1 h at room temperature with appropriate secondary antibodies i.e., Alexa Fluor 488-conjugated anti-mouse IgG (1:100) or Cy3-conjugated donkey anti-rabbit IgG (1:500), both from Jackson Immuno-Research Laboratories. Nuclear staining was achieved using mounting medium containing DAPI (Cat #0100-20, DAPI Fluoromount-G, Southern Biotech). Images were acquired using an Olympus FluoView confocal laser-scanning microscope.

### 4.7. Subcellular Fractionation and Immunoblotting

TM cells were grown in 100-mm tissue culture plates. Twenty-four hours after seeding, growth medium was replaced with fresh DMEM medium supplemented with 10% EV-free fetal calf serum, containing either EVS-N or EVS-OS at the suitable concentration (100-fold higher than TM cells amount). After 8 and 24 h incubation, cell nucleus and cytoplasmic fractions were separated as previously described (doi:10.12659/MSM.894467). TM cells were washed with PBS buffer on ice, scraped into lysis buffer [20 mM HEPES (pH 7.4), 1 mM EGTA, 1 mM EDTA, 10% glycerol, 1 mM Na_3_VO_4_, 1 mM MgCl_2_, 25 mM NaF, 150 mM NaCl] supplemented with complete protease inhibitor mixture, homogenized at 25,000 rpm on a Polytron (PT 1200, Kinematica AG, Switzerland) for 1 min and centrifuged at 600× *g*, 15 min, 4 °C. The nuclear-free supernatant was further centrifuged at 20,000× *g* for 30 min, 4 °C to collect the cytosolic fraction. The nuclear pellet was re-suspended in 0.25 M sucrose containing 10 mM MgCl_2_ and protease inhibitor mixture and was laid on 0.88 M sucrose containing 0.5 mM MgCl_2_ and protease inhibitor mixture. The sucrose gradient was centrifuged at 2800× *g* for 10 min, 4 °C. The nuclear pellet was re-suspended in RIPA buffer [50 mM Tris (pH 7.5), 150 mM NaCl, 1% NP-40, 0.5% deoxycholate, protease inhibitors], sonicated for 50 s (Sonicator ultrasonic processor, Misonix Inc., Farmingdale, NY, USA) and centrifuged again at 2800× *g* for 10 min, 4 °C. Protein concentrations were measured using the Bradford assay (Bio-Rad Laboratories, Hercules, CA, USA). Equal amount of cell fractions were resolved on a 10% SDS-PAGE, Western blotted and probed with Nrf2 (1:200, ab62352, Abcam), β-catenin (1:1000, D10A8, Cell Signaling), phospho-GSK3β (1:1000, 5B3, Cell Signaling), β-actin (1:4000, A2228, Sigma-Aldrich) and lamin B antibodies (1:1000, sc-6216, Santa Cruz).

### 4.8. Protein Concentration of TM Lysates

The total protein concentrations were determined according to the Bradford method, with BSA as a standard [42]. Briefly, standard solutions were diluted with Bradford regent (Bio-Rad Laboratories, Hercules, CA, USA), and the mixture of the two was incubated at room temperature for 5 min. The absorbance was read at 595 nm on a spectrophotometer (Microplate reader model 680, Bio-Rad) and then a standard curve was obtained. The samples protein level was assessed according to this curve.

### 4.9. Real-Time Quantitative Polymerase Chain Reaction (qRT-PCR)

Total RNA was isolated from TM cells 8 h after incubation with EVS-N or EVS-OS by using EZ-RNA Kit (Cat# 20-400-100, Biological Industries Ltd., Beit Haemek, Israel) according to the manufacturer’s instructions. RNA quantitation was performed by measuring the absorbance of the RNA sample solutions at 260 nm using a BioDrop Spectrophotometer (BioDrop, Cambridge, UK). Total RNA (1 μg) was used for generation of cDNA with a qScript commercial kit (Quanta Biosciences, Gaithersburg, MD, USA) in a 20-μL reaction according to the manufacturer’s protocol. Real-time PCR reactions were conducted using Power SYBR Green Master Mix (Life Technologies) on an Applied Biosystems Real-Time PCR 7500 system (Applied Biosystems). Thermal cycling conditions used in this study were as follows: 95 °C for 10 min, followed by 40 cycles of 95 °C for 15 s, and 60 °C for 1 min. The related mRNA levels were normalized to the 18S mRNA level. Results were analyzed using 7500 Software v 2.0.4 (Applied Biosystems). The primers used in this study are listed in Appendix B.

### 4.10. ROS Measurements

The TM cells were plated in black 96-well plates at a density of 5 × 10^3^ cells/well. After overnight incubation, cells were washed with cold PBS buffer and incubated with EVS-N or EVS-OS or remained untreated (control) at 37 °C in 5% CO_2_ incubator. Twenty-four hours after treatment, cells were further incubated with 20 µM ROS measurements probe 2′,7′-dichlorofluorescin diacetate (DCFDA) (Cat# D6883, Sigma, St. Louis, MO, USA) for 1 h. The solution was aspirated, and the cells washed with PBS. This was followed by the addition of 50 µL PBS and an additional 50 µL of PBS containing 300 µM AAPH. The fluorescence intensity of DCFDA was determined immediately at 15 min intervals over a period of 3 h at 37 °C. Fluorescence were read by a microplate reader (Infinite M200, Tecan, Switzerland) at an excitation wavelength of 492 nm and emission wavelength of 525 nm.

### 4.11. TM Antioxidant Enzyme Activities

For the determination of SOD and catalase (CAT), the TM cells were seeded in 6 mm sterile culture dishes (1 × 10^6^) for 24 h. Thereafter, the cells were co-incubated with EVs released by NPCE cells treated with or without 15 mM AAPH. Twenty-four hours post-treatment cells were washed ×3 with PBS buffer, scraped with rubber policeman, and pelleted by centrifugation for 5 min at 200× *g*, 4 °C. The pellet containing TM cells was re-suspended in 50 Mm phosphate buffer, pH 7.8 and sonicated on ice at 40% amplitude for 60 s. The SOD activity of the TM cells was measured spectrophotometrically by using nitroblue tetrazolium (NBT) method as described by Beyer and Fridovich [43]. The reaction mixture contained 50 mM phosphate buffer (pH 7.8), 14 mM methionine, 75 μM NBT, 4 μM riboflavin, 0.1 mM EDTA, and 10 μL extracted TM proteins. The reaction mixtures were illuminated for 10 min and the photo-reduction of NBT (formation of purple formazan) was measured at 560 nm using microplate reader (model 680; Bio-Rad). SOD activity was determined using a calibration curve from 1.2 to 10 SOD units/mL.

The CAT activity was measured by the modified method of Cohen et al. Fifty microliters of extracted proteins and 800 µL of phosphate buffer (pH 7.0) were combined. The reaction was initiated by the addition of 100 µL of the stock 60 mM H2O2, followed by gentle mixing. At 10 min, 100 µL aliquots were withdrawn and quenched in solution containing 4.0 mL of 0.6 N H2SO4 and 1 mL of 10 mM FeSO4. The color of the products formed was developed by the addition 400 µL of 2.5 M KSCN, and the absorbance values of the red color of ferrithiocyanate product were calorimetrically recorded at 460 nm. The standard curve of known concentrations of pure CAT was used to calculate the absolute values of test samples.

### 4.12. Statistical Analysis

Experimental data are presented as the mean ± SEM. The statistical analyses were performed using the GraphPad Prism version 5 software (GraphPad Software, Inc., La Jolla, CA, USA). Tests of significance were conducted by one-way analysis of variance (ANOVA), followed by post hoc multiple comparison test (Tukey—Kramer Multiple Comparison Test) and analysis of Western Blot experiments was carried out using a two-way ANOVA followed by Bonferroni post-test. Statistical significance was considered at *p* < 0.05. Carbonyl assay results were analyzed using two tails *t*-test, significance was considered at *p* < 0.05.

## Figures and Tables

**Figure A1:**
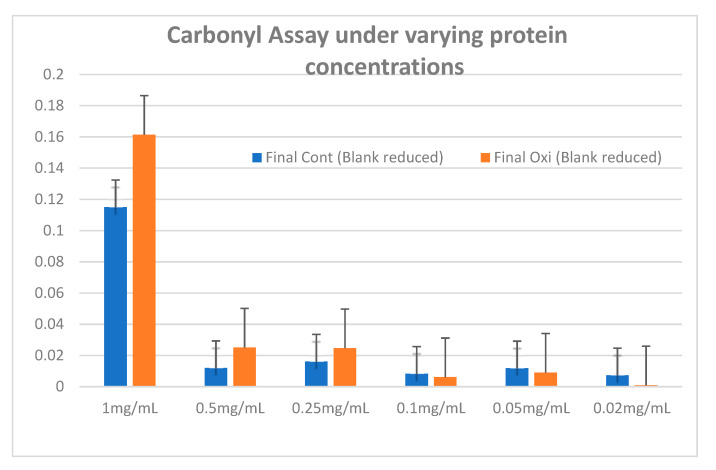
Carbonylated proteins in control and oxidized NPCE cells (15 mM AAPH, 90 min) were measured at 366 nm using the DNPH method. Data are represented by mean ± SD.

## Appendix A

To assess the sensitivity of the carbonyl assay to the protein concentration found in the example, we conducted a series of experiments to find the lower limit at which it would be possible to differentiate between proteins that have undergone OS to the control group.

## Appendix B

**Table A1:** The sequence of primers used in qRT-PCR.

Gene	Forward Primer	Reverse Primer
*Sod1*	5′-GGTGGGCCAAAGGATGAAGAG-3′	5′-CCACAAGCCAAACGACTTCC-3′
*Sod2*	5′-GCTCCGGTTTTGGGGTATCTG-3′	5′-GCGTTGATGTGAGGTTCCAG-3′
*Gpx1*	5′-CAGTCGGTGTATGCCTTCTCG-3′	5′-GAGGGACGCCACATTCTCG-3′
*Hmox1*	5′-AAGACTGCGTTCCTGCTCAAC-3′	5′-AAAGCCCTACAGCAACTGTCG-3′
*Nrf2*	5′-TCAGCGACGGAAAGAGTATGA-3′	5′-CCACTGGTTTCTGACTGGATGT-3′
*18S*	5′-ATCCCTGAAAAGTTCCAGCA-3′	5′-CCCTCTTGGTGAGGTCAATG-3′
